# Performance of Bamboo Bark Fiber Asphalt Mortar Modified with Surface-Grafted Nano-SiO_2_

**DOI:** 10.3390/polym16192850

**Published:** 2024-10-09

**Authors:** Nan Zhang, Xichen Wang, Pei Sun, Nanxiang Zheng, Aodi Sun

**Affiliations:** 1 School of Urban Construction and Transportation, Hefei University, Hefei 230601, China; zhangnan@hfuu.edu.cn (N.Z.); 17351027541@163.com (X.W.); sunp@hfuu.edu.cn (P.S.); 19966546848@163.com (A.S.); 2 Anhui Provincial Key Laboratory of Urban Rail Transit Safety and Emergency Management, Hefei University, Hefei 230601, China; 3 Key Laboratory for Special Region Highway Engineering of Ministry of Education, Chang’an University, Xi’an 710064, China

**Keywords:** bamboo bark fiber, asphalt mortar, modification method, modification mechanism, nano-SiO_2_

## Abstract

In this study, the feasibility of using bamboo bark fibers as modifiers to enhance asphalt mortar performance was investigated. Bamboo bark fibers were modified with NaOH, KH570 silane coupling agent, and nano-SiO_2_, and their preparation methods were established. The modified fibers were assessed for their oil absorption, thermal stability, and hydrophobicity. The asphalt mortar was evaluated for three key indicators: rutting resistance, deformation resistance, and durability at high temperatures. The microscopic morphology and modification mechanisms of the fibers were also studied. The results showed that modification with NaOH increased fiber porosity and surface roughness, while KH570 and its hydrolysis products enabled nano-SiO_2_ grafting onto the fibers, improving their adsorption to asphalt. The NaOH-KH570-nano-SiO_2_ ternary-composite-modified bamboo bark fiber (NKSBF) demonstrated superior hydrophobicity, oil absorption, and thermal stability at the asphalt mixing temperature. Among the modified fibers, asphalt mortar containing 3% NKSBF showed the best performance based on three key indicators, increased the shear strength by 96.4% and the softening point by 7.1% compared to the base asphalt, and increased the ductility by 1% compared to lignin fiber asphalt mortar. The incorporation of 3% bamboo bark fibers improved the rutting resistance, deformation resistance, and durability of short-term-aged asphalt mortar, with NKSBF showing the most significant improvement.

## 1. Introduction

To address the rising traffic volume and prolong the service life of asphalt pavement, researchers have explored the incorporation of fiber materials into asphalt and its mixtures in recent years to enhance their performance. Findings indicate that the inclusion of fibers significantly improves the high-temperature stability, low-temperature cracking resistance, and water stability of asphalt mortar and its mixtures [1,2,3,4]. Currently, synthetic and mineral fibers are increasingly used in asphalt; however, they are associated with high costs and environmental impacts related to their production. Consequently, researchers have increasingly focused on plant fibers, which are renewable, cost-effective, lightweight, and environmentally friendly, in recent years [5,6,7]. Common plant fibers utilized as asphalt modifiers include lignin fibers, rice straw fibers, cotton stalk fibers, and corn stalk fibers, the majority of which are derived from plant waste. By processing these waste materials into fibers and incorporating them into asphalt and its mixtures, significant enhancements in road performance can be achieved, thus providing an effective solution for the rational utilization of plant waste [8,9,10,11,12]. As a major agricultural nation, China generates substantial plant waste. Utilizing plant waste to produce plant fibers for asphalt modification not only improves asphalt pavement performance but also mitigates the environmental pollution caused by plant waste, aligning with China’s dual carbon development goals. In numerous plant fibers, bamboo fiber exhibits exceptional performance. Compared to other plant fibers, its high tensile strength, toughness, and thermal stability provide it with a significant advantages in asphalt modification [13,14]. Currently, bamboo materials are widely utilized in furniture, crafts, and civil construction. The processing of bamboo generates substantial waste, which is often incinerated. Converting this waste into bamboo fibers for use in asphalt is anticipated to mitigate waste accumulation [15]. Furthermore, compared to lignin fibers derived from slowly growing natural wood, the rapid-growth characteristic of bamboo makes bamboo fibers an effective alternative for alleviating wood supply issues [16]. Jia et al. [17,18] investigated the effects of bamboo fiber on Superpave asphalt mixtures, revealing that incorporating 0.3% bamboo fiber significantly enhances permanent deformation and crack resistance at medium temperatures, as well as stiffness and fatigue resistance under long-term aging. Sheng et al. [19] demonstrated that 0.2% to 0.4% bamboo fiber markedly improves rutting and low-temperature crack resistance in stone matrix asphalt (SMA) mixtures. Furthermore, Ahmed et al. [20] reported that the Superpave asphalt mixture with 0.3% bamboo fiber exhibits superior performance metrics, such as Marshall stability, indirect tensile strength, and rut depth, compared to those with the same proportion of bagasse fiber. The above research indicates that incorporating bamboo fibers into asphalt and its mixtures can effectively enhance their durability and resistance to cracking, thereby improving overall performance. This approach promotes the effective utilization of renewable resources, reduces reliance on traditional wood, alleviates environmental pressure, and demonstrates promising socioeconomic benefits and development prospects.

Bamboo fiber exhibits excellent performance; however, compared to synthetic and mineral fibers, it has lower hydrophobicity and thermal stability [21,22]. Research indicates that the surface modification of plant fibers can enhance the adhesion performance between the fibers and the composites by altering their surface morphology, reducing surface polarity, or introducing additional chemical groups [23]. Therefore, surface modification is necessary to enhance bamboo fiber’s performance in asphalt applications. Currently, surface modification techniques for plant fibers are categorized into two main types: physical and chemical [23,24,25,26]. The physical methods encompass heat treatment and steam explosion, whereas the chemical methods include acetylation, silane coupling agent treatment, and alkali treatment. Among the various methods, the most widely adopted and effective treatments for plant fibers are silane coupling agents and alkali treatments, which enhance the water stability, low-temperature cracking resistance, high-temperature stability, and durability of the interfacial bonding with asphalt [27,28,29,30,31,32]. Cui et al. [22] applied surface modifications to bamboo fibers using NaOH and KH570 silane coupling agents. The modifications resulted in rougher surfaces and increased hydrophobicity, which subsequently enhanced the resistance of bamboo fiber asphalt mortar to deformation at high temperatures. Similarly, Li et al. [33] conducted an investigation into the effects of NaOH treatment on the performance of bamboo fibers in asphalt. The study revealed that NaOH treatment significantly enhanced fiber dispersion, improved high-temperature stability, increased resistance to low-temperature cracking and fatigue in asphalt mixtures, and strengthened the interfacial adhesion between bamboo fibers and asphalt. These studies suggest that the effectiveness of bamboo fibers in asphalt can be significantly enhanced by treating the fibers with KH570 silane coupling agents and NaOH. However, the effects of combined modifications remain unclear, as prior research on bamboo fibers has primarily focused on individual modification techniques. Xie et al. [34] found that, compared to NaOH treatment alone, a composite modification of bagasse fibers with NaOH and KH570 silane coupling agents resulted in improved hydrophobicity and thermal stability. To thoroughly investigate the extent of performance enhancement and the underlying modification mechanisms, it is essential to conduct composite modifications of bamboo fibers using NaOH and KH570 silane coupling agents and compare the results with those of individual modifications.

Modification with NaOH and silane coupling agents enhances the performance of plant fibers in asphalt but simultaneously reduces the oil absorption capacity of the fibers [27]. As a composite material modifier, nano-SiO_2_ effectively improves the high-temperature stability, water resistance, fatigue resistance, and aging resistance of asphalt due to its large specific surface area, high surface activity, and excellent thermal stability when directly added to asphalt [35,36,37,38]. However, this modification method requires substantial amounts of nano-SiO_2_, making it economically inefficient. Currently, there is no research on grafting nano-SiO_2_ onto plant fibers’ surfaces as a modification method and improving asphalt performance by incorporating the modified fibers. Thus, in this study, a small amount of nano-SiO_2_ was utilized in combination with NaOH and KH570 silane coupling agents for the ternary composite modification of bamboo fibers, chemically grafting nano-SiO_2_ onto the bamboo fibers’ surfaces. This approach significantly reduces the required amount of nano-SiO_2_. The nano-SiO_2_ grafted onto the fiber surface is expected to effectively fill the microcracks and defects between the fibers and asphalt, improving interfacial bonding and thereby enhancing the fibers’ oil absorption capacity and other asphalt properties. Additionally, the approach used in this study innovatively utilizes discarded bamboo bark—which possesses superior mechanical properties—from bamboo plywood production as the raw material for preparing bamboo fibers, replacing the conventional method that uses bamboo’s inner structure [39]. This approach not only achieves waste utilization but also effectively conserves resources. Through hydrophobicity, oil absorption, and thermal stability tests, as well as scanning electron microscopy (SEM) and Fourier transform infrared spectroscopy (FTIR) analysis, the modification mechanisms of bamboo bark fibers at different stages and their bonding performance with asphalt were systematically investigated. Bamboo bark fiber-modified asphalt mortars were subjected to penetration, softening point, and ductility tests to determine the optimal fiber content. Additionally, the durability, high-temperature rutting resistance, and deformation resistance of bamboo bark fiber asphalt mortars were evaluated using a dynamic shear rheological (DSR) test and a multiple stress creep recovery (MSCR) test. A comparative analysis with lignin-fiber-modified asphalt mortars was conducted to comprehensively assess the modification effects of bamboo bark fibers. As lignin fibers are commonly used as a benchmark, they help provide a deeper understanding of the potential applications of bamboo bark fibers in asphalt.

## 2. Materials and Methods

### 2.1. Materials

**Figure 1:**
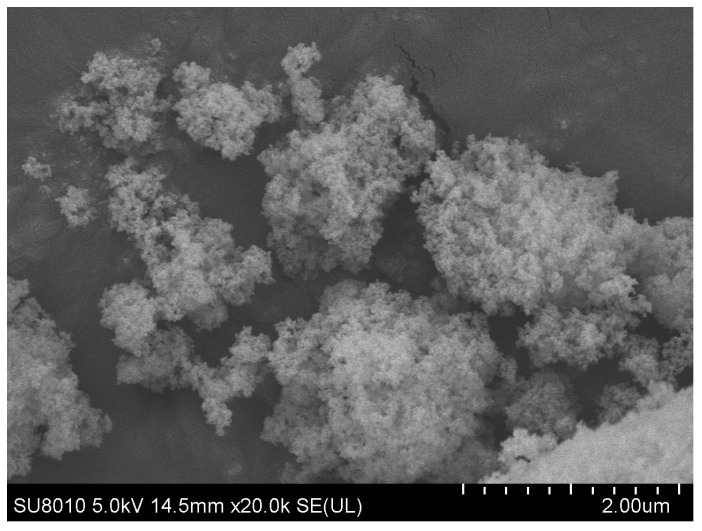
Microscopic morphology of nano-SiO_2_.

Bamboo bark with a thickness of 1 mm, discarded from the production of bamboo plywood, was processed into the bamboo bark fibers used in the experiments. Table 1 provides the key performance parameters of the lignin fibers supplied by Chuangsheng Building Materials Technology Co., Ltd. (Handan City, China), as well as the parameters of the bamboo bark fibers. The modifiers used included nano-SiO_2_, NaOH solution, and KH570 silane coupling agent, all supplied by Zhenhan New Materials Co., Ltd. (Nanjing, China). The performance parameters and microstructure of nano-SiO_2_ are presented in Table 2 and Figure 1. Grade 70 asphalt from Sinopec Maoming Company (Maoming, China). was used as the base asphalt in the trials. Table 3 lists the main technical indicators of the asphalt.

### 2.2. Preparation of Fibers and Asphalt Mortars

#### 2.2.1. Preparation of Bamboo Bark Fibers

**Figure 2:**
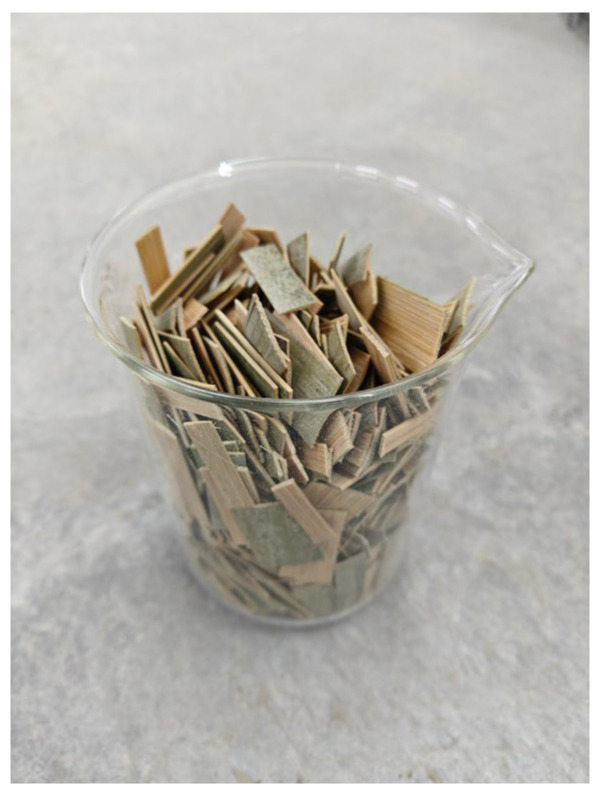
Cut finished bamboo bark.

**Figure 3:**
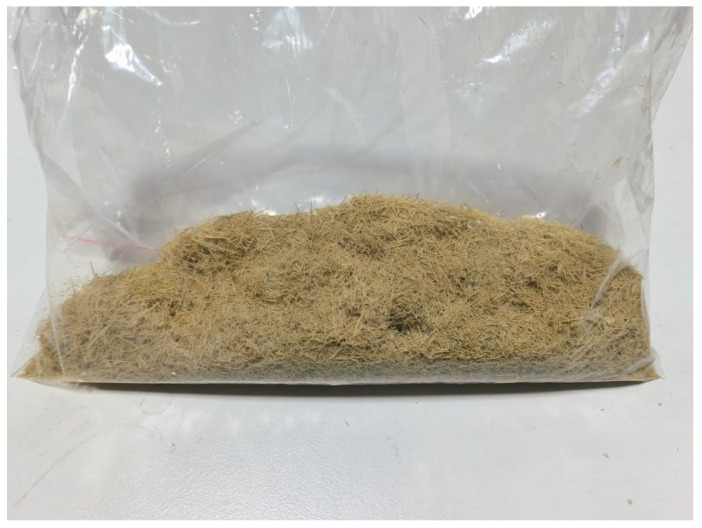
Prepared and screened bamboo bark fibers.

The bamboo bark was initially cut into 3 cm long slices (Figure 2) and then subjected to wet treatment by soaking it in distilled water for 24 h to facilitate softening. Subsequently, the bamboo bark was placed in a sealed crusher and crushed for 1 min to obtain bamboo bark fibers, which were then dried in an oven at 80 °C until the fibers reached a constant weight. Afterward, the fibers were sieved to select those of the appropriate length. Previous research has indicated that the bamboo fibers used for asphalt modification to enhance road performance typically range between 4 and 8 mm in length [17,18,20,40,41]. However, shorter fibers are more prone to poor dispersion within the asphalt, leading to clumping and entanglement, which can negatively affect the asphalt’s road performance. To mitigate this issue, bamboo bark fibers between 6 and 8 mm in length were selected through sieving to be used as asphalt modifiers in this experiment. Figure 3 illustrates the prepared and screened bamboo bark fiber products.

#### 2.2.2. Preparation of Modified Bamboo Bark Fibers

**Figure 4:**
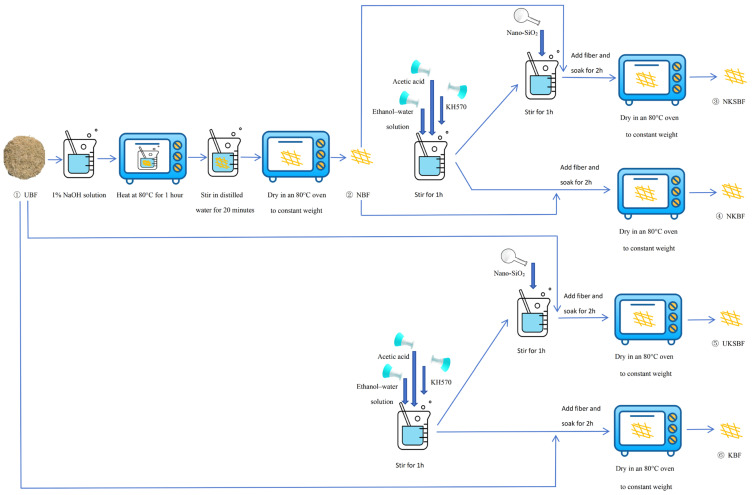
Preparation process of modified bamboo bark fibers.

Figure 4 illustrates the procedure for preparing the modified bamboo bark fiber samples. First, the bamboo bark fibers were submerged in a 1% NaOH solution by mass and reacted at 80 °C in an oven for 1 h. Following the reaction, the fibers were stirred in distilled water for 20 min, filtered to remove residual liquid, and then dried in an oven at 80 °C until they reached a constant weight. Subsequently, KH570, with a quantity equal to 3% of the fiber mass, was added to an ethanol–water solution in a 9:1 volume ratio, and the pH of the mixture was adjusted to 3–4 by adding acetic acid. The mixture was then agitated for 1 h to produce the KH570 solution. Nano-SiO_2_, representing 2% of the fiber mass, was then incorporated into the mixture. After stirring for 1 h, the NaOH-modified bamboo bark fibers were thoroughly soaked in the solution and allowed to stand at room temperature for 2 h. Finally, the fibers were dried to a constant weight in an oven at 80 °C, resulting in NaOH-KH570-nano-SiO_2_ ternary composite-modified bamboo bark fibers (NKSBFs). Additionally, various bamboo bark fiber modifications were prepared to investigate their effects on the properties of bamboo bark fiber asphalt mortar, including unmodified bamboo bark fibers (UBFs), KH570-modified bamboo bark fibers (KBFs), KH570-nano-SiO_2_-modified bamboo bark fibers (UKSBFs), NaOH-modified bamboo bark fibers (NBFs), and NaOH-KH570-modified bamboo bark fibers (NKBFs).

#### 2.2.3. Preparation of Asphalt Mortar

Fiber-modified asphalt mortars were produced using a high-speed shear mixer and a magnetic heating stirrer equipped with temperature control, with fiber contents accounting for 1%, 2%, 3%, and 4% of the asphalt mass. Throughout the preparation process, the temperature of the asphalt mortar was maintained at 160 °C.

### 2.3. Microstructure and Fiber Composition Tests

#### 2.3.1. Scanning Electron Microscopy (SEM)

To examine the microscopic characteristics of bamboo bark fibers, a SU8010 cold-field emission scanning electron microscope (HITACHI, Tokyo, Japan) was employed to analyze the fiber microstructure at 500× and 2000× magnifications.

#### 2.3.2. Fourier Transform Infrared Spectroscopy (FTIR)

Fourier transform infrared spectroscopy was utilized to qualitatively analyze the fiber composition. An infrared spectrometer was used for this purpose, with a scanning range from 4000 to 500 cm^−1^, and a total of 32 scans were performed at a resolution of 4 cm^−1^.

### 2.4. Basic Fiber Performance Tests

#### 2.4.1. Hydrophobicity

The hydrophobicity of the fibers was evaluated based on the contact angle between the fibers and water. This contact angle was measured using the sessile drop method. First, the bamboo bark fibers were crushed and sieved through a 0.075 mm mesh to produce bamboo bark fiber powder. This powder was then modified following the procedure outlined in Figure 4 and dried at 80 °C until it reached a constant weight. The modified and unmodified powders were separately spread onto microscope slides, ensuring uniformity by pressing the powder layer to a consistent thickness of 1 mm. Water droplets were placed on the prepared surfaces, and the contact angles were recorded with a camera at the 5 s mark. Finally, the captured images were digitized and analyzed using the SDC-350 contact angle measuring instrument, manufactured by Kunshan Shengding Industrial Intelligent Technology Co., Ltd. (Jiangsu Province, China), to determine the contact angles.

#### 2.4.2. Oil Absorption

The oil absorption of fibers is characterized by the oil absorption rate, a crucial performance indicator for road fibers. The magnitude of this rate significantly influences the fibers’ effectiveness in enhancing the adhesion and toughness of asphalt. In this experiment, a basket drip test was employed to determine the oil absorption rate of the fibers. The procedure involved mixing 50 g of fully fluid asphalt with 5 g of fibers and placing the mixture into an iron pan with a mesh size of 0.18 mm. The mass of the empty iron pan was recorded as m_1_. The iron pan was then heated at a constant temperature of 160 °C for 2 h, and, every 0.5 h, the total mass of the iron pan and the dripping asphalt was measured and recorded as m_2_. Finally, the oil absorption rate X of the fibers at different heating times was calculated using Formula (1):

(1) $$X=\frac{m_{2}-m_{1}}{50}\times 100\%$$

#### 2.4.3. Thermal Stability

A 5 g sample of bamboo bark fibers was heated continuously at 160 °C for 5 h. To assess the thermal stability of the fibers at the temperature used during asphalt mixture preparation, the mass loss rate was measured hourly.

### 2.5. Fiber-Reinforced Asphalt Mortar Performance Tests

#### 2.5.1. Cone Penetration

**Figure 5:**
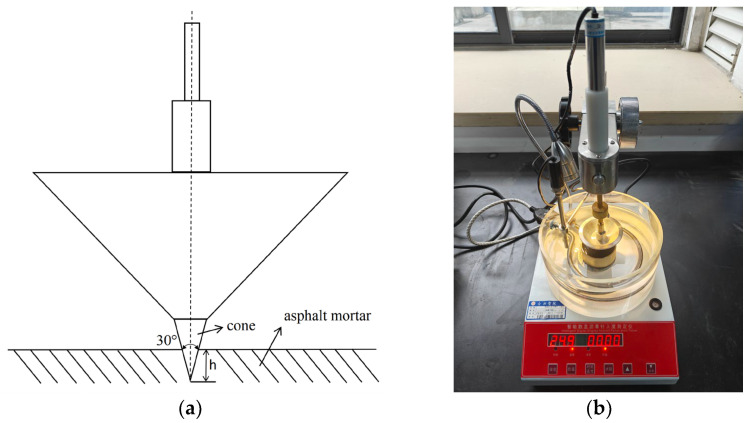
The cone penetration test of asphalt mortar: (**a**) a schematic diagram of the cone; (**b**) a picture of the test.

Fiber asphalt mortar is a heterogeneous material; therefore, conventional penetration tests are not suitable for accurately characterizing its ability to resist shear failure. To address this, a cone penetration test was employed to assess this property. The IV-2000 asphalt penetration tester, manufactured by Tuozhan Instrument Equipment Co., Ltd. (Shanghai, China), measured the cone penetration of the fiber asphalt mortar, and Formula (2) was used to calculate the material’s shear strength. The test cone and rod, with a combined mass of 150.15 g, had a 30° cone tip angle and a 5 s penetration time, and tests were conducted at a temperature of 25 °C. Figure 5a shows a schematic diagram of the cone, while Figure 5b displays an image of the cone penetration test.

(2) $$\tau =\frac{981Q\cos^{2}\left(\frac{\alpha }{2}\right)}{\pi h^{2}\tan\left(\frac{\alpha }{2}\right)}$$

In this equation, τ is the shear strength (MPa); Q is the penetration mass (the sum of the masses of the weights, cone needle, and connecting rod) (150.15 g); h is the penetration depth (0.1 mm); and α is the angle of the cone tip (30°).

#### 2.5.2. Softening Point and Ductility

The softening point and ductility of asphalt mortar characterize its high-temperature stability and low-temperature cracking resistance performance, respectively. To determine these properties, the softening point of the fiber asphalt mortar was first measured using an asphalt softening point tester, applying a heating rate of 5 °C per minute. Subsequently, the ductility of the fiber asphalt mortar was assessed at 10 °C using a low-temperature asphalt ductility tester.

#### 2.5.3. Dynamic Shear Rheological (DSR) Test

A HAAKE MARS 60 rheometer (Thermo Scientific, Waltham, MA, USA) was used to perform a dynamic shear rheological test of the asphalt samples containing bamboo bark fibers. The effect of bamboo bark fibers on the resistance of asphalt mortars to high-temperature rutting was evaluated using three parameters: the complex shear modulus (G*), rutting factor (G*/sinδ), and phase angle (δ). The asphalt mortar sample was 1 mm thick, the parallel plate mold had a diameter of 25 mm, and the testing frequency was 10 rad/s, with the temperature adjusted in 6 °C increments from 46 °C to 82 °C.

#### 2.5.4. Multiple Stress Creep Recovery (MSCR)

To replicate the short-term aging behavior experienced by asphalt mortar during mixing and laying, asphalt mortar samples initially underwent aging through the rolling thin-film oven test (RTFOT) method, as outlined in JTG E20-2011 [42]. Subsequently, the multiple stress creep recovery (MSCR) test was conducted. A Thermo HAAKE MARS 60 rheometer was utilized to perform continuous tests at two creep stress levels: 0.1 kPa and 3.2 kPa. Each stress level included ten creep–recovery cycles, each consisting of 1 s of creep loading and 9 s of recovery. The total test duration was 300 s. The deformation recovery rate (R) and non-recoverable creep compliance (J_nr_) were used to evaluate the high-temperature deformation resistance and durability of the bamboo bark fiber-modified asphalt mortar. Formulas (3) and (4) were applied to calculate R and J_nr_, respectively:

(3) $$R=\frac{\gamma_{p}-\gamma_{\mathrm{nr}}}{\gamma_{p}-\gamma_{0}}$$

(4) $$J_{\mathrm{nr}}=\frac{\gamma_{\mathrm{nr}}-\gamma_{0}}{\tau }$$

In these equations, γ_p_ is the maximum strain within each loading cycle; γ_nr_ is the residual strain within each loading cycle; γ_0_ is the initial strain within each loading cycle; and τ is the shear stress.

## 3. Results

### 3.1. Microstructure and Composition of Modified Bamboo Bark Fibers

#### 3.1.1. Micro-Topography

**Figure 6:**
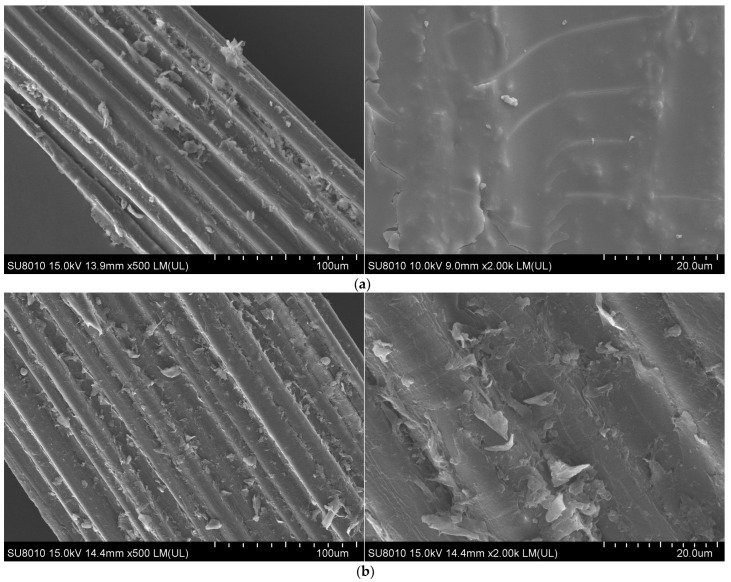

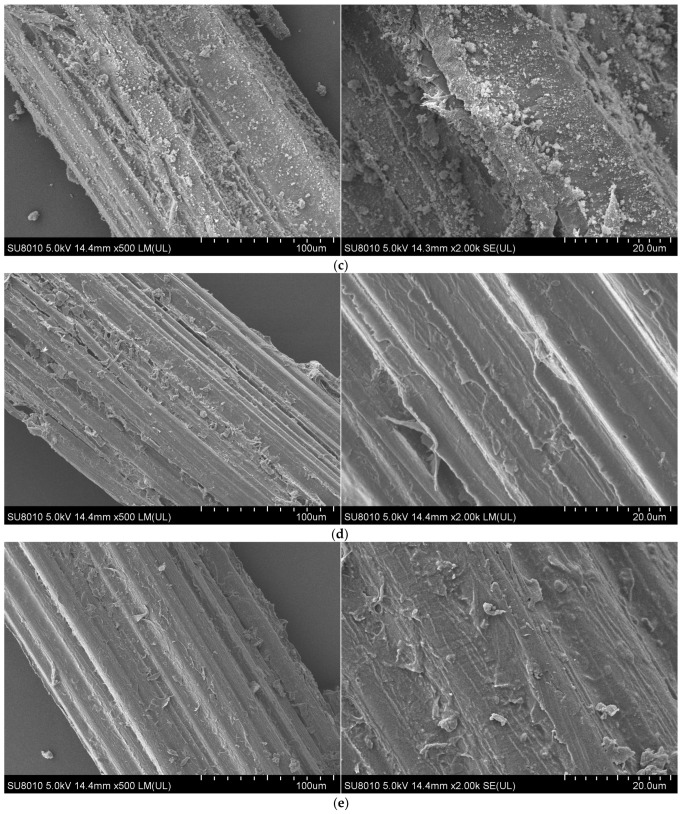

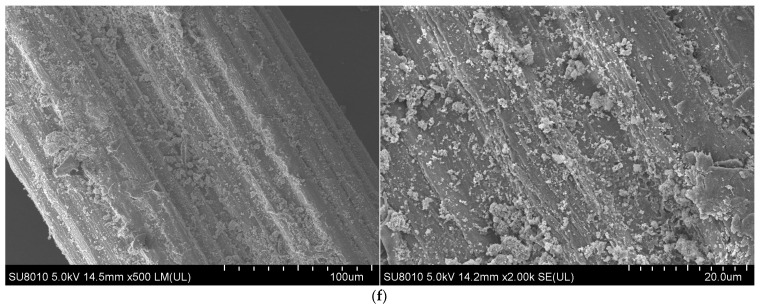
Microscopic morphology of bamboo bark fibers before and after modification: (**a**) UBF, (**b**) KBF, (**c**) UKSBF, (**d**) NBF, (**e**) NKBF, (**f**) NKSBF.

Using a scanning electron microscope, the morphological properties of the six samples—UBF, KBF, UKSBF, NBF, NKBF, and NKSBF—were analyzed, as shown in Figure 6.

Modification using NaOH, KH570, and nano-SiO_2_ enhanced the surface roughness and specific surface area of the bamboo bark fibers, as shown in Figure 6a–f. Compared to other modified fibers, the surface of the UBFs was smoother and is covered with a layer of wax, ash, and some impurities (Figure 6a). Modification with NaOH alone eliminated surface impurities, ash, and the wax layer. The surface roughness of the fibers increased considerably, resulting in more noticeable grooves and cracks and exposing the internal tubular structure (Figure 6d). Modification with KH570 alone further increased the number of wrinkles on the fiber surface and enhanced its roughness (Figure 6b). After modification with NaOH and KH570, the internal tubular structures and surface cracks were filled, but the roughness increased due to additional surface wrinkles (Figure 6e). Subsequent modification with nano-SiO_2_, following either single or combined modifications with NaOH and KH570, resulted in a significant number of SiO_2_ particles adhering to the fiber surface. As a result, the specific surface area and roughness of the bamboo bark fibers further increased, enhancing their ability to adsorb asphalt (Figure 6c,f).

#### 3.1.2. FTIR Analysis

**Figure 7:**
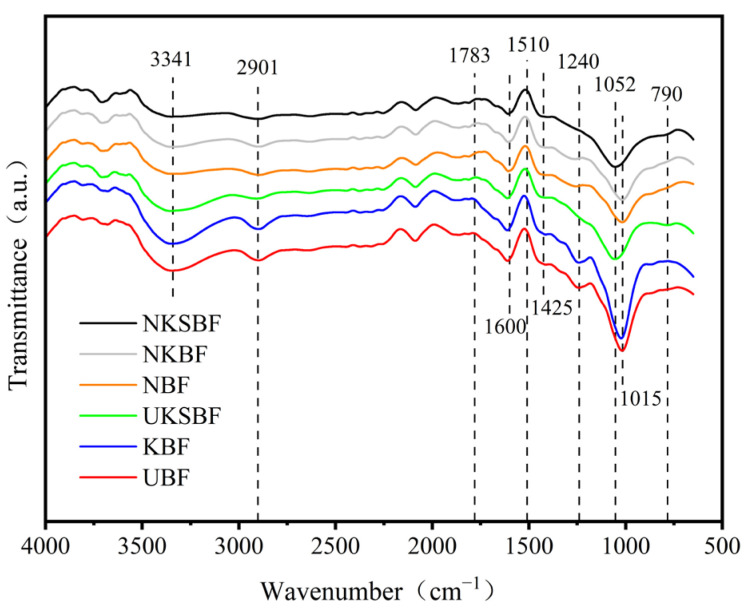
Infrared spectra of bamboo bark fibers before and after modification.

The six samples were analyzed qualitatively using Fourier transform infrared spectroscopy: UBF, KBF, UKSBF, NBF, NKBF, and NKSBF. The results are presented in Figure 7.

**Figure 8:**
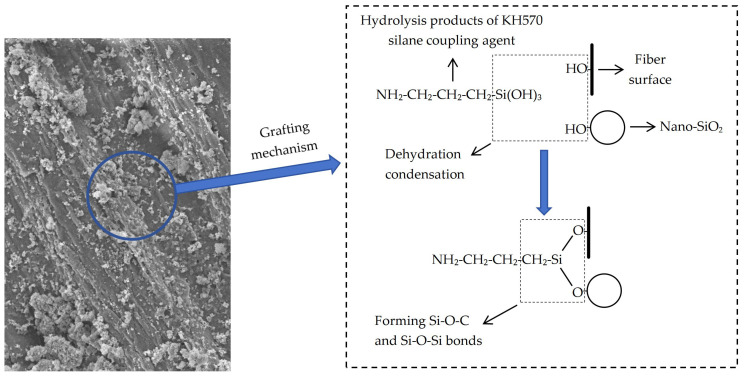
Mechanism of grafting nano-SiO_2_ onto bamboo bark fiber surface.

The absorption peak observed at approximately 3341 cm^−1^ for both modified and unmodified bamboo bark fibers can be attributed to the –OH stretching vibrations in lignin, cellulose, and hemicellulose, as shown in Figure 7 [43]. NaOH-induced alkaline hydrolysis eliminated the wax layer, ash, contaminants, and some cellulose, hemicellulose, and lignin with a high -OH content from the fiber surface, resulting in a weaker absorption peak for NBF than for UBF [44,45]. The peak at 2901 cm^−1^ is associated with the stretching vibrations of –CH groups in the carbon chains of cellulose and hemicellulose [46]. The wavelength shift in the absorption peak intensity of NBF is similar to that at 3341 cm^−1^ due to the reduced number in –CH groups resulting from the decreases in cellulose and hemicellulose after NaOH modification. The absorption bands of aromatic rings are represented by peaks at 1600 cm^−1^, 1510 cm^−1^, and 1425 cm^−1^, indicative of lignin [47]. The intensities of these three characteristic lignin peaks decreased somewhat after NaOH modification. This indicated that NaOH is more effective than lignin in breaking down cellulose and hemicellulose, as evidenced by the more notable decrease observed at 2901 cm^−1^. The absorption peak at 1728 cm^−1^ is associated with the stretching vibrations of C=O groups in RCOOR and ROR crosslinks between cellulose and hemicellulose [48]. The peak at 1240 cm^−1^ is associated with the C–O bond stretching vibrations of the acetyl groups of hemicellulose [49]. The absorption peaks at 1783 cm^−1^ and 1240 cm^−1^ for NBF, NKBF, and NKSBF were weaker than those for UBF, KBF, and UKSBF. This reduction was attributed to the substantial presence of hemicellulose and cellulose on the bamboo bark fiber surface, which partially degraded during NaOH modification, resulting in decreased numbers of C=O and C-O groups. The distinct and intense absorption peak at 1015 cm^−1^ is associated with the C−O stretching vibrations in cellulose, hemicellulose, and lignin or the C−O−C stretching vibrations in cellulose and hemicellulose [50]. NaOH modification decreased the levels of cellulose, hemicellulose, and lignin, resulting in a reduced absorption peak for NBF at this wavelength. The characteristic Si–O–Si stretching vibration peak at 790 cm^−1^ in UKSBF and NKSBF can be attributed to the formation of Si–O–Si bonds through the dehydration condensation of Si–OH, which is generated by the hydrolysis of KH570 and -OH groups on the surface of nano-SiO_2_ [27]. The Si-O-C absorption peak at 1052 cm^−1^ indicates that nano-SiO_2_ modification facilitates the reaction between Si–OH and hydroxyl groups on the bamboo bark fiber surface, resulting in the creation of Si–O–C bonds [51]. This mechanism is illustrated in Figure 8, which depicts how KH570 covalently grafts nano-SiO_2_ onto bamboo bark fibers.

KH570 alone did not significantly impact the intensity of any absorption peaks of bamboo bark fibers when used for modification. Its primary role in the modification process was to facilitate the grafting of nano-SiO_2_ onto bamboo bark fibers. Modification with nano-SiO_2_ reduced all characteristic absorption peaks of the bamboo bark fibers. This indicated that nano-SiO_2_, grafted onto the bamboo bark fibers through KH570, adhered closely to and covers the fiber surface, thereby effectively improving the interfacial compatibility between the bamboo bark fibers and asphalt.

### 3.2. Basic Performance of Modified Bamboo Bark Fibers

#### 3.2.1. Hydrophobicity Analysis

**Figure 9:**
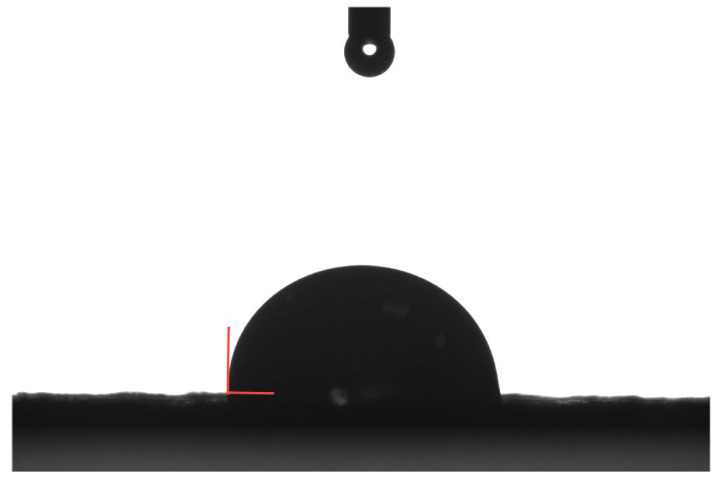
A schematic of the fiber–water contact angle.

**Figure 10:**
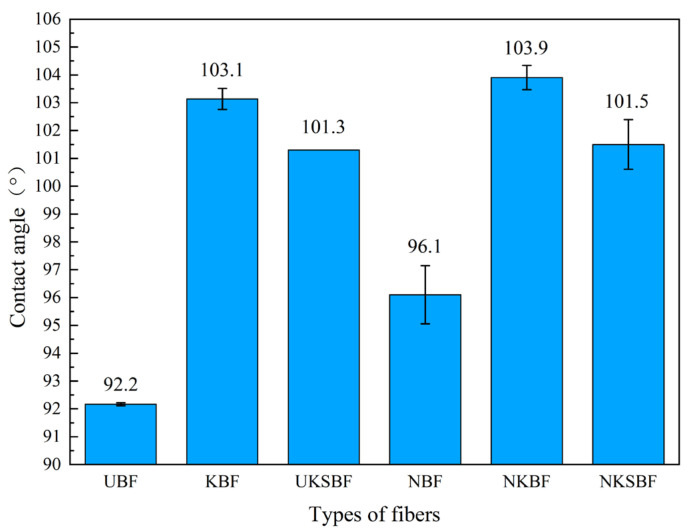
Contact angles of different fibers with water.

The contact angle between the fibers and water serves as an indicator of fibers’ hydrophobic properties; a larger contact angle signifies enhanced hydrophobicity and a stronger bonding performance with asphalt [52]. Figure 9 shows a schematic diagram of the contact angles between the fibers and water, while Figure 10 presents the measured contact angles of the different fibers with water.

Figure 10 shows that the contact angles of modified bamboo bark fibers with water were higher than those of UBF. The contact angles of the NKBF, KBF, NKSBF, UKSBF, and NBF were higher by 12.7%, 11.8%, 10.1%, 9.9%, and 4.2%, respectively. KH570 modification enhanced the contact angle of bamboo bark fibers with water more effectively than NaOH modification. After modification with nano-SiO_2_, the contact angle of bamboo bark fibers decreased slightly. This observation, combined with the FTIR analysis, indicated that NaOH modification degrades cellulose, hemicellulose, and lignin on the fiber surface, reducing –OH groups and decreasing the fibers’ water absorption capacity. However, it also weakened the ash, contaminants, and wax layer, which prevent moisture absorption, resulting in only a slight increase in the contact angle. KH570 modification enhanced the hydrophobicity of the fibers significantly by forming chemical bonds with the –OH groups on the fiber surface and fully filling the pores and cracks within the fibers. Due to the inherent hydrophilicity of nano-SiO_2_, the KH570 solution did not fully react with it during the preparation of the modifier. Consequently, some nano-SiO_2_ surfaces were not completely bonded with KH570 hydrolysis products through chemical bonds, leading to a slight reduction in hydrophobicity. Among the modified fibers, although the contact angle of NKSBF with water was lower than those of KBF and NKBF, the difference was minimal, and NKSBF’s hydrophobic performance remained satisfactory.

#### 3.2.2. Oil Absorption Analysis

**Figure 11:**
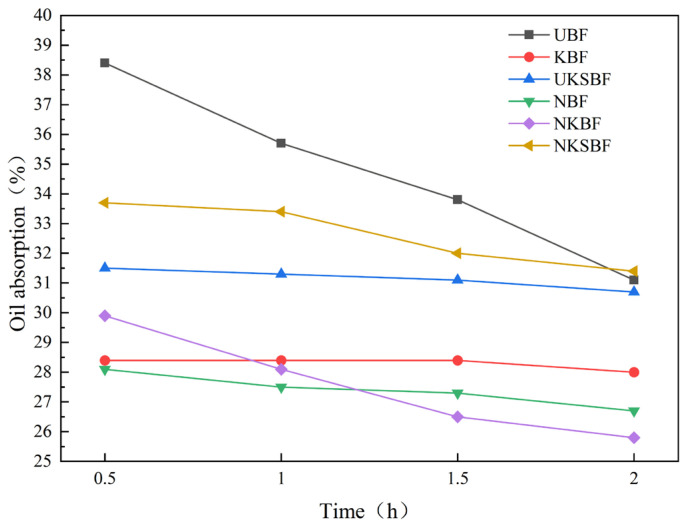
Variation in oil absorption rate of bamboo bark fibers, before and after modification, with time at 160 °C.

The oil absorption of the fibers was assessed based on their oil absorption rate. A higher oil absorption rate indicates a greater capacity of the fibers to retain asphalt, reflecting a more significant improvement in the adhesion between the fibers and the asphalt [53]. Figure 11 illustrates the oil absorption rates of bamboo bark fiber asphalt mortar, measured for 2 h at a constant temperature of 160 °C.

Figure 11 shows that UBFs initially exhibited the highest oil absorption rate. The oil absorption rates of the bamboo bark fibers, both before and after modification, decreased steadily with increasing heating time. Among them, UBF demonstrated the most significant reduction, indicating that at the mixing temperature, they had lower bonding stability with asphalt. After a 2 h heating process at 160 °C, the oil absorption rates of the fibers were ranked as follows: NKSBF > UBF > UKSBF > KBF > NBF > NKBF. Notably, the oil absorption rate of NKSBF was 0.9% higher than that of UBF, whereas those of KBF, NBF, and NKBF were 10%, 14.2%, and 17% lower than that of UBF, respectively. This suggests that after modification with NaOH and KH570, the oil absorption rate of bamboo bark fibers decreases significantly, whereas it increases after grafting with nano-SiO_2_. The initially high oil absorption rate of UBF is attributed to its wax layer, impurities, and ash, which can absorb a substantial amount of asphalt [27]. However, after treatment with NaOH and KH570, the ash, impurities, and wax layer on the fiber surface either disappear or become covered, reducing the fibers’ capacity to retain asphalt. Compared to modified bamboo bark fibers, UBF exhibited a more significant decrease in the oil absorption rate as a result of the detachment of some of the wax layer, impurities, and ash on its surface during the longer heating period. The significant increase in the oil absorption rate following grafting with nano-SiO_2_ indicates that nano-SiO_2_ possesses a high capacity to retain asphalt. This enhancement in retention capacity further improves the adhesion between the bamboo bark fibers and the asphalt.

#### 3.2.3. Thermal Stability Analysis

**Figure 12:**
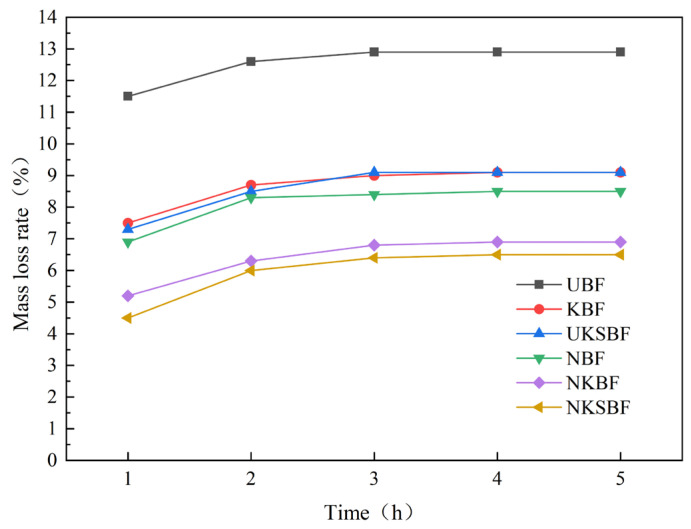
Variation in mass loss rate of bamboo bark fibers, before and after modification, with time at 160 °C.

The mass loss rate characterizes the thermal stability of the fibers at the mixing temperature of asphalt mixtures. A lower mass loss rate suggests that the fibers are less prone to brittle failure or carbonization, thereby enhancing performance stability [52]. The mass loss rate of bamboo bark fibers was monitored over a period of 5 h at a constant temperature of 160 °C, as illustrated in Figure 12.

Figure 12 demonstrates that the modified bamboo bark fibers exhibited superior thermal stability compared to UBF, as evidenced by their lower mass loss rates. At all observed time points, NKSBF displayed the lowest mass loss rate. After a 5 h heating period at 160 °C, the mass loss rates of the fibers were ranked in the order UBF > KBF = UKSBF > NBF > NKBF > NKSBF, with NKSBF showing a 49.6% reduction in mass loss rate compared to UBF. Except for NBF, the mass loss rates of the fibers followed a similar pattern over time, increasing steadily for 3 h and then plateauing. The mass loss rate of NBF remained stable after 2 h, primarily because NaOH treatment removed the wax layer, impurities, and portions of cellulose and hemicellulose from the bamboo bark fiber surface, which are prone to decomposition at high temperatures [22,33]. This treatment also increased the surface porosity and cracks of the fibers; within the first 2 h of high-temperature heating, the bound water inside the fibers evaporated rapidly. Based on the micro-topography and FTIR analyses, it was found that after modification with KH570 and nano-SiO_2_, the pores and cracks on the fiber surface were filled through chemical grafting with KH570 and a substantial amount of nano-SiO_2_, effectively blocking the evaporation of bound water. As a result, the fibers exhibited better thermal stability throughout the entire heating period.

### 3.3. Performance of Asphalt Mortar with Modified Bamboo Bark Fibers

#### 3.3.1. Shear Strength

**Figure 13:**
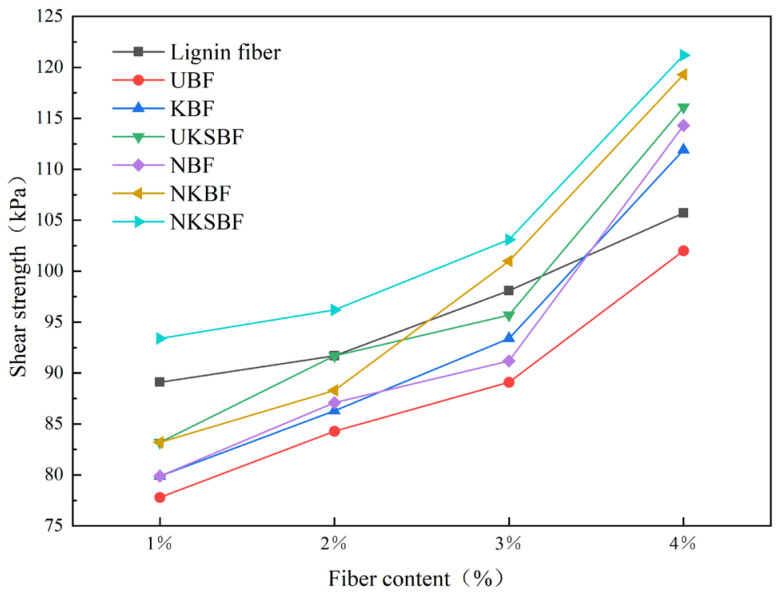
The variation in the shear strength of various fiber asphalt mortars with increasing fiber content.

The shear strength of an asphalt mortar indicates its ability to resist shear failure. A higher shear strength signifies a greater ability to resist shear failure. Figure 13 illustrates the variation in the shear strength of the bamboo bark fiber asphalt mortar and lignin fiber asphalt mortar before and after modification at different fiber contents. As shown in Figure 13, the shear strength of asphalt mortar increased with increasing fiber content across all fiber types. Compared to UBF, the modified bamboo bark fiber asphalt mortars exhibited a significant increase in shear strength, with NKSBF asphalt mortar demonstrating the highest shear strength. For asphalt mortars with a 3% fiber content, the shear strengths exceeded those of UBF by 4.8%, 7.4%, 2.4%, 13.4%, and 15.7% for KBF, UKSBF, NBF, NKBF, and NKSBF, respectively. These results indicate that the incorporation of nano-SiO_2_ and the application of NaOH and KH570 modification techniques effectively enhance the shear strength of bamboo bark fiber asphalt mortar. This improvement is primarily attributed to NaOH modification, which increases the roughness and porosity of the bamboo bark fiber surfaces, thereby enhancing their ability to mechanically interlock with the asphalt. Consequently, the asphalt mortar becomes more viscous and exhibits better shear strength. Additionally, the hydrolysis products of KH570 can form siloxane bridges with the functional groups in the asphalt, chemically bonding the bamboo bark fibers to the asphalt and further enhancing its shear strength [22]. The high surface activity of nano-SiO_2_ allows for more thorough interaction with asphalt molecules. Due to its very small particle size, nano-SiO_2_ can also fill the tiny pores at the fiber–asphalt interface, improving interfacial properties and significantly enhancing shear strength.

#### 3.3.2. Softening Point

**Figure 14:**
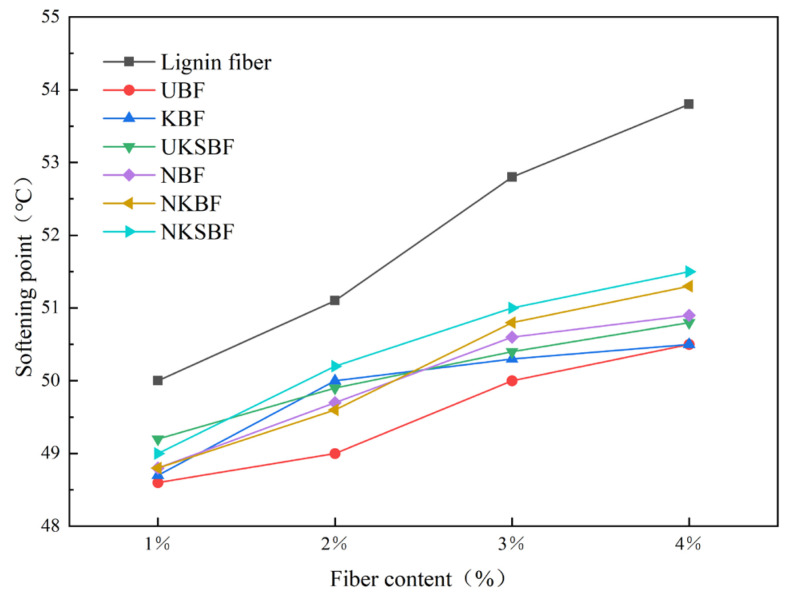
The variation in the softening point of various fiber asphalt mortars with increasing fiber content.

The softening point reflects the thermal stability of asphalt mortar. An elevated softening point denotes improved performance at high temperatures. Figure 14 illustrates the variation in the softening points of the bamboo bark fiber asphalt mortar and lignin fiber asphalt mortar before and after modification at different fiber contents. The modified bamboo bark fiber asphalt mortar exhibited a higher softening point compared to UBF, although it remained lower than that of the lignin fibers, as shown in Figure 14. Among the modified samples, the NKSBF asphalt mortar had the highest softening point. Compared to UBF, the softening points increased by 0.8%, 2.5%, 2%, and 2% at fiber contents of 1%, 2%, 3%, and 4%, respectively. The softening point of the asphalt mortar progressively rose with increasing fiber content. The increase was more pronounced when the fiber content was raised from 1% to 3%, while the growth rate slowed when the content increased from 3% to 4%. This phenomenon could be attributed to the formation of a stable network structure of fibers within the asphalt material once the fiber content reached a certain threshold. The effect of additional fiber content on this network structure diminished beyond this point. The higher softening point of lignin fibers is primarily due to their shorter length and narrower width, which result in a larger specific surface area for the same mass compared to bamboo bark fibers [54]. This increased specific surface area enables more asphalt to adhere to the fiber surface, enhancing the viscosity of the asphalt mortar. Modifications involving NaOH, KH570, and nano-SiO_2_ effectively raised the softening point of the bamboo bark fiber asphalt mortar, aligning with the observed improvements in shear strength.

#### 3.3.3. Ductility

**Figure 15:**
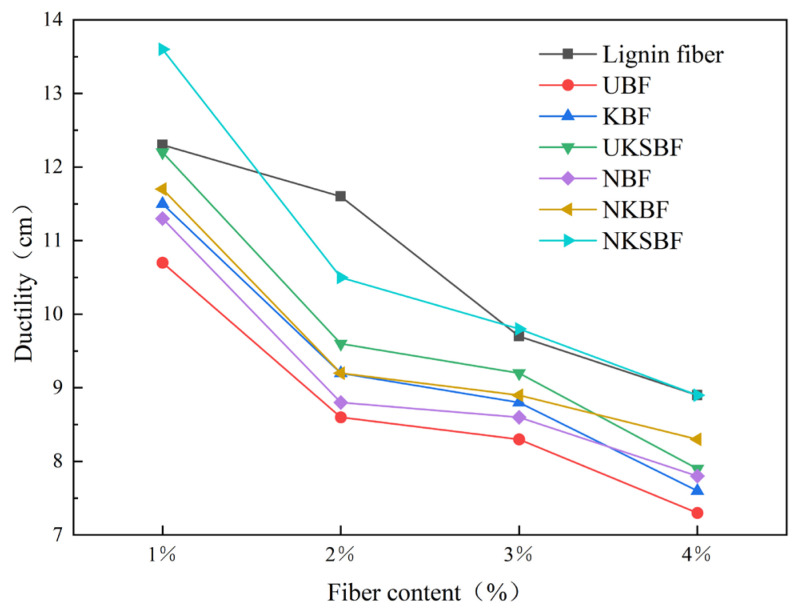
The variation in the ductility of various fiber asphalt mortars with increasing fiber content.

The ductility of asphalt mortar indicates its low-temperature cracking resistance. A higher ductility signifies better resistance to cracking at low temperatures. Figure 15 illustrates the variation in the ductility of the bamboo bark fiber asphalt mortar and lignin fiber asphalt mortar before and after modification at different fiber contents. Figure 15 demonstrates that as the fiber content increased, the ductility of the asphalt mortars gradually decreased. This decline was primarily due to the introduction of defects by the fibers into the continuous structure of the asphalt, transforming it from a “continuum” to a “discontinuum”. Stress concentrations occur at these fiber–asphalt interface defects during tensile testing, potentially causing ruptures and reducing ductility. At a fiber content of 1%, there were fewer fibers and thus fewer interface defects between the fibers and the asphalt. As the fiber content increased to 2%, the number of overlaps rose, leading to insufficient bonding between the fibers and the asphalt, which resulted in stress concentrations and a significant decrease in ductility. A 3% fiber content caused a smaller reduction in ductility as the increased overlaps formed a spatial network structure among the fibers that could partially withstand stress [55]. However, as the fiber content continued to increase beyond this point, the excessive number of fibers introduced numerous defects into the asphalt mortar, further decreasing the material’s ductility. The modified bamboo bark fiber asphalt mortar exhibited higher ductility than the UBF asphalt mortar, with NKSBF asphalt mortar displaying the highest ductility. Compared to UBF, the ductility increased by 27.1%, 22.1%, 18.1%, and 21.9% at fiber contents of 1%, 2%, 3%, and 4%, respectively. The primary reason for this improvement was the NaOH modification, which enhanced ductility by roughening the surfaces of the bamboo bark fibers and improving their mechanical interlocking with the asphalt. Further improvement in the ductility of the asphalt mortar was achieved through KH570 and nano-SiO_2_ grafting modifications, which effectively reduced the interface defects between the fibers and the asphalt.

#### 3.3.4. Optimal Fiber Dosage

The ideal fiber content for bamboo bark fiber asphalt mortar was 3% of the asphalt mass, based on a comprehensive analysis of Figure 13, Figure 14 and Figure 15, which compare the shear strength, softening point, and ductility of the mortar before and after modification at different fiber contents. At this content, these properties were ranked as follows: for shear strength, NKSBF > NKBF > lignin fibers > UKSBF > KBF > NBF > UBF; for the softening point, lignin fibers > NKSBF > NKBF > NBF > UKSBF > KBF > UBF; and, for ductility, NKSBF > lignin fibers > UKSBF > NKBF > KBF > NBF > UBF. Among the modified bamboo bark fiber asphalt mortars, NKSBF demonstrated the best performance, with increases of 96.4% and 7.1% in the shear strength and softening point, respectively, compared to the base asphalt. The ductility of NKSBFs was 1% higher than that of lignin fibers, although it remained lower than that of the base asphalt.

#### 3.3.5. Dynamic Shear Rheological (DSR) Characteristics

**Figure 16:**
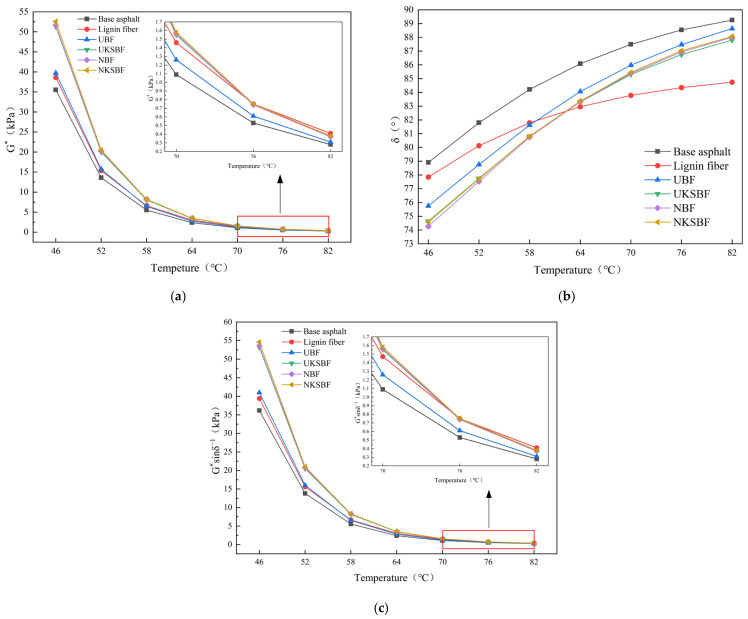
Variation in (**a**) complex shear modulus, (**b**) phase angle, and (**c**) rutting factor of base asphalt and different fiber asphalt mortars with increasing temperature.

Based on the results of the tests got fiber oil absorption, thermal stability, and hydrophobicity, as well as the shear strength, softening point, and ductility of the fiber asphalt mortars, UKSBF and NKSBF asphalt mortars with 3% fiber content, which demonstrated superior performance, were selected for dynamic shear rheological testing. These were compared with UBF, NBF, lignin fiber, and base asphalt to assess the effects of various modification techniques on the high-temperature rutting resistance of the asphalt mortars. Figure 16 illustrates the variations in the complex shear modulus, phase angle, and rutting factor with increasing temperature for both the base asphalt and the different fiber asphalt mortars.

The complex shear modulus and rutting factor reflect the ability of asphalt mortar to resist rutting at high temperatures. Higher complex shear modulus and rutting factor signify better high-temperature rutting resistance. As illustrated in Figure 16a,c, the incorporation of fibers enhanced the complex shear modulus and rutting factor of the asphalt mortar, thus improving its high-temperature rutting resistance. Compared to the lignin fiber asphalt mortar, UBF exhibited a higher complex shear modulus and rutting factor at lower temperatures. Nonetheless, as the temperature increased, the difference between them diminished. Modifying bamboo bark fibers further improved the complex shear modulus and rutting factor of asphalt mortar at various temperatures, with NKSBF showing the most significant overall enhancement. In contrast to lignin fibers, bamboo bark fibers, due to their longer length, more effectively interlock in asphalt to form a spatial network structure at lower temperatures, providing structural support and resulting in higher complex shear modulus and rutting factor [56]. However, as the temperature rises, the increased asphalt flow destabilizes the network structure and reduces its load-bearing capacity, thereby diminishing the asphalt mortar’s high-temperature rutting resistance [34]. On the other hand, lignin fibers, with their shorter length and smaller diameter, do not readily form a spatial network structure in asphalt. However, their higher specific surface area and denser distribution compared to bamboo bark fibers lead to greater-viscosity asphalt mortar, resulting in good rutting resistance at elevated temperatures [52]. As demonstrated in Figure 10, Figure 11 and Figure 12, although the load-bearing capacity of the spatial network structure of bamboo bark fibers modified with NaOH, KH570, and nano-SiO_2_ did not improve, the interface bonding performance between the fibers and asphalt was significantly enhanced, leading to notable increases in the complex shear modulus and rutting factor of the modified fibers. At 82 °C, although the complex shear modulus and rutting factor of the modified bamboo bark fiber asphalt mortar were slightly lower than those of lignin fibers, they were still markedly greater compared to that of the base asphalt and UBF, demonstrating excellent high-temperature rutting resistance.

The phase angle, a key indicator, reflects the ratio and interaction between the viscous and elastic components in asphalt. A smaller phase angle indicates stronger elastic properties of asphalt mortar, meaning that deformation is more easily recoverable after unloading [57,58]. As shown in Figure 16b, the phase angles of the base asphalt and all fiber-modified asphalt mortars gradually increased with temperature, indicating that the viscous components rose while the elastic components decreased. The phase angles of the lignin fiber and UBF asphalt mortars were significantly lower than that of the base asphalt at various temperatures, demonstrating that the incorporation of fibers effectively enhanced the elastic components in asphalt. At 46 °C, UBF asphalt mortar’s phase angle was notably lower than that of the lignin fibers, but as the temperature rose, the difference between them progressively diminished. Modifying bamboo bark fibers effectively reduced the phase angle of asphalt mortar at different temperatures. This observation aligns with the principles underlying the trends observed in the complex shear modulus and rutting factor.

#### 3.3.6. Multiple Stress Creep Recovery (MSCR)

**Figure 17:**
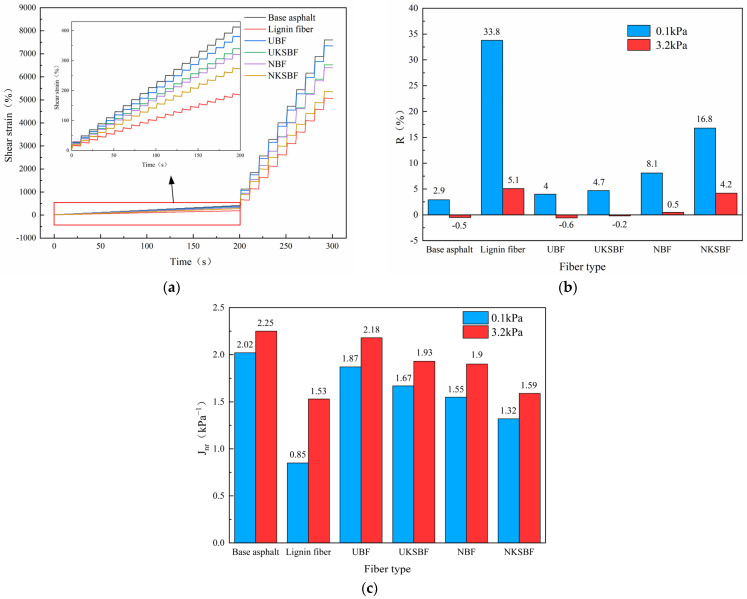
(**a**) Creep–recovery curves, (**b**) deformation recovery rate, and (**c**) non-recoverable creep compliance for base asphalt mortar and various fiber-modified asphalt mortars.

Figure 17 presents the creep–recovery curves for the base asphalt and various fiber-modified asphalt mortars after short-term aging, as well as the non-recoverable creep compliance (J_nr_) at 0.1 and 3.2 kPa and the deformation recovery rate (R).

The creep–recovery curves, deformation recovery rates, and non-recoverable creep compliance collectively indicate the deformation resistance and durability of asphalt mortar at high temperatures. Lower shear strains and non-recoverable creep compliance, along with higher deformation recovery rates, signify better performance in resisting deformation and ensuring durability at high temperatures. The shear strains of all short-term-aged asphalt mortars, as shown in Figure 17a, were considerably lower at the 0.1 kPa stress level compared to the 3.2 kPa stress level. This indicates that the pavement’s resistance to deformation at high temperatures is more influenced by increases in vehicle load [52]. Additionally, the shear strains of the bamboo bark and lignin fibers were lower than those of the base asphalt at various stress levels, both before and after modification. This suggests that even during the short aging period that occurs during mixing and paving, the fibers enhance the asphalt mixture’s durability and resistance to deformation at high temperatures.

Both the modified and unmodified bamboo bark fibers enhanced the deformation recovery rate of the short-term-aged asphalt mortar at the 0.1 kPa stress level, as illustrated in Figure 17b. Among these, NKSBF showed the most significant improvement, with the deformation recovery rate being 534.6% higher compared to that of the base asphalt, though they remained less effective than lignin fibers. All asphalt mortars exhibited a notable decrease in deformation recovery rates at the 3.2 kPa stress level. In particular, the recovery rate of the short-term-aged base asphalt dropped to negative values, indicating extremely poor recovery. Adding UBF had a minimal impact on the recovery rate, whereas modifying the fibers with NaOH or grafting nano-SiO_2_ to them significantly improved it. The asphalt’s spatial network structure, composed of overlapping bamboo bark fibers, supported recovery at the 0.1 kPa stress level [22]. However, this network was disrupted at the higher stress level of 3.2 kPa, hindering deformation recovery during unloading. Based on the FTIR analysis, NaOH modification removed the hard wax layer, impurities, and ash from the bamboo bark fibers’ surfaces and partially breaks down cellulose, hemicellulose, and lignin, increasing fiber porosity and enhancing fiber elasticity, thereby improving deformation recovery. Owing to its exceptionally small particle size and high surface activity, nano-SiO_2_ filled gaps and microcracks between the asphalt and fibers, enhancing the mechanical properties at the interface and facilitating stress transmission, which further improved deformation recovery. Figure 17c demonstrates that incorporating bamboo bark fibers reduced the non-recoverable creep compliance in the asphalt mortar, which improved high-temperature deformation resistance [59]. Furthermore, the NaOH and nano-SiO_2_ modifications further reduced non-recoverable creep compliance. The non-recoverable creep compliance values were ranked as follows at stress levels of 0.1 and 3.2 kPa: base asphalt > UBF > UKSBF > NBF > NKSBF > lignin fibers. Among the modified bamboo bark fibers, NKSBFs showed the greatest improvement; however, they still did not perform as well as lignin fibers. Compared to the base asphalt, the non-recoverable creep compliance of NKSBFs was lower by 34.7% and 29.3% at the 0.1 and 3.2 kPa stress levels, respectively.

## 4. Conclusions

In this study, the microscopic morphology, modification mechanisms, and fundamental properties of bamboo bark fibers modified by means of nano-SiO_2_ grafting and various other modification methods were investigated. The performance of the resulting asphalt mortar was evaluated based on three key indicators: high-temperature rutting resistance, deformation resistance, and durability. The following conclusions were drawn:

(1) The surface roughness and specific surface area of the bamboo bark fibers were enhanced following modification with NaOH, KH570 silane coupling agent, and nano-SiO_2_. The hydrolysis of KH570 produced Si–OH groups, which subsequently condensed with the -OH groups on the bamboo bark fibers and nano-SiO_2_ to form Si–O–C and Si–O–Si bonds. This covalent bonding improved the attachment of nano-SiO_2_ to the fibers and subsequently enhanced their asphalt adsorption performance.
(2) At an asphalt mixture mixing temperature of 160 °C, modifications with NaOH and KH570 reduced the oil absorption of bamboo bark fibers, whereas grafting with nano-SiO_2_ increased it. The NKSBFs exhibited the highest oil absorption, with a 0.9% increase compared to UBF after 2 h of heating. The modified fibers demonstrated more stable asphalt adsorption performance. NaOH modification significantly improved thermal stability, and KH570 modification and nano-SiO_2_ grafting also enhanced it. After 5 h of heating, NKSBF showed the best thermal stability, with a 49.6% reduction in the mass loss rate compared to UBF.
(3) NaOH modification slightly improved the hydrophobicity of the fibers, and KH570 modification significantly enhanced it, whereas grafting with nano-SiO_2_ slightly reduced the hydrophobicity due to the incomplete reaction between KH570 and nano-SiO_2_. Nevertheless, the hydrophobic performance remained strong. NKSBF exhibited a 10.1% higher water contact angle compared to UBF, indicating enhanced hydrophobicity and reduced water absorption.
(4) Incorporating both modified and unmodified bamboo bark fibers enhanced the shear strength and softening point of asphalt mortar but reduced its ductility. The modified fibers exhibited superior performance, with a 3% content being optimal. At this concentration, the NKSBF asphalt mortar demonstrated a 96.4% increase in shear strength and a 7.1% increase in the softening point compared to the base asphalt, with its ductility surpassing that of lignin fibers by 1%.
(5) At a 3% fiber content, the UBF asphalt mortar showed significantly better high-temperature rutting resistance compared to the base asphalt across all tested temperatures. UBF also outperformed lignin fiber asphalt mortar at lower temperatures. However, at higher temperatures, the lignin fiber asphalt mortar outperformed UBF. NKSBF exhibited the best overall high-temperature rutting resistance, although it was slightly lower than that of the lignin fibers at the highest temperature.
(6) Bamboo bark fibers at a 3% content significantly improved the high-temperature deformation resistance and short-term aging durability of asphalt mortar. NaOH modification and nano-SiO_2_ grafting further enhanced these improvements. Among the modified fibers, NKSBFs exhibited the greatest enhancement, with reductions in non-recoverable creep compliance of 34.7% and 29.3% at stress levels of 0.1 kPa and 3.2 kPa, respectively, compared to the base asphalt.

## Figures and Tables

**Table 1:** Performance parameters of lignin fibers and bamboo bark fibers.

Material Name	Length, mm	Color	Ash Content, %	pH	Density, g/cm^3^
Lignin fibers	1 (Average)	Gray	18.1	7.2	1.28
Bamboo bark fibers	6–8	Brown-yellow	13.7	7.0	1.33

**Table 2:** Performance parameters of nano-SiO_2_.

Material Name	Mean Particle Size, nm	Specific Surface Area, m^2^/g	Bulk Density, g/L	pH
nano-SiO_2_	20	240	150	7.5

**Table 3:** The main technical indicators of asphalt.

Tested Property	Detection Result	Standard
Penetration (25 °C), 0.1 mm	67	60~80
Cone penetration (25 °C), 0.1 mm	55.8	Measured value
Shear strength, kPa	52.5	Measured value
Softening point, °C	47.6 ≥ 46	
Ductility (10 °C), cm	24	≥15
Dynamic viscosity (60 °C), Pa·s	231	≥180
Wax content (distillation method), %	2	≤2.2
Flash point, °C	292	≥260
Solubility, %	99.81	≥99.5
Density (15 °C), g/cm^3^	1.032	Measured value

## Data Availability

Data are contained within the article.
